# Hypokalemia Induced Partial Nephrogenic Diabetes Insipidus: A Case Report

**DOI:** 10.31729/jnma.8501

**Published:** 2024-03-31

**Authors:** Anil Nepali, Prakriti Adhikari, Amit Shah, Shailes Paudel, Prakriti Bhandari

**Affiliations:** 1 Intensive Care Unit, Patan Academy of Health Sciences, Lagankhel, Lalitpur, Nepal; 2 Department of Internal Medicine, Dhulikhel Hospital, Dhulikhel, Kavre, Nepal

**Keywords:** *case reports*, *diabetes insipidus*, *Gitelman syndrome*, *hypokalemia*, *nephrogenic*

## Abstract

Diabetes insipidus is a condition characterised by a large volume of diluted urine production and increased thirst. In this case report, a 49-year-old gentleman presented with 3 months of polyuria and polydipsia. He had a repeated history of hypokalemia. On the evaluation of polyuria and polydipsia, he was diagnosed with partial nephrogenic diabetes insipidus based on his inability to concentrate urine after a water deprivation test and his less than 50% response to exogenous desmopressin. On the evaluation of recurrent hypokalemia, the investigation reports met biochemical criteria for the diagnosis of Gitelman syndrome. He was encouraged to increase his fluid intake as required, and potassium chloride supplementation relieved his symptoms. This case report demonstrates the reversibility of nephrogenic diabetes insipidus with a correction of hyperkalemia.

## INTRODUCTION

Diabetes insipidus (DI) comprises central and nephrogenic forms, related to Arginine Vasopressin (AVP) dysfunction.^1^ Central DI results from reduced AVP production, while nephrogenic DI stems from impaired kidney response to AVP.^1,2^ Gitelman syndrome (GS), is a rare tubulopathy with a prevalence of 1-10 per 40,000 induces electrolyte imbalances, with hypokalemia potentially causing reversible nephrogenic DI.^2,3,4^ The polyuria and polydipsia spectrum encompasses central DI, nephrogenic DI, primary polydipsia, and gestational DI, each involving distinct mechanisms of AVP regulation or responsiveness.^1^ We hereby report a rare case of partial nephrogenic diabetes insipidus linked to hypokalemia, fully reversed upon potassium correction.

## CASE REPORT

A 49-year-old gentleman presented with excessive thirst and polyuria for 3 months, particularly more during the night. He used to wake up at night due to excessive thirst and had nocturia 3-4 times. His estimated urine output was more than 5 litres per day. He had a history of repeated hospital visits with signs and symptoms of hypokalemia, for which he was managed with oral potassium supplements and dietary counselling. He had no history of headaches or vision problems. He was a non-smoker and non-alcoholic and had not used any medication or recreational drugs. He had no history of any chronic illness like diabetes mellitus, chronic renal failure, or any psychiatric illness. There was no history of similar illness in the family.

On physical examination, his vital signs were within normal limits. Initial blood investigations revealed low serum potassium levels (1.8 mEq/dl), high serum sodium levels (154 mEq/dl), low serum magnesium levels (1.5 mg/dl), high plasma osmolarity (312 mosm/kg), normal serum random glucose levels (114 mg/dl), normal Glycosylated haemoglobin (HbA1c) levels (5.9%), normal urine osmolarity (347 mosm/kg), serum copeptin levels (24 pmol/L). Also, urine calcium creatinine ratio was sent and found to be 0.15 mmol/mmol. Renal and hepatic function tests were done and found to be normal. An abdominal ultrasound showed bilateral normal kidneys.

The patient underwent a water deprivation test. After 8 hours of water deprivation, his plasma osmolarity increased from 312 mosm/kg to 321 mosm/day, and his urine osmolarity increased from 347 mosm/kg to 422 mosm/kg. Intramuscular desmopressin (2 micrograms) was administered, following which urine osmolarity increased to 499 mosm/kg. Serum potassium and magnesium were low, and a urine calcium to creatinine ratio of 0.09 mmol/mmol raised the suspicion of Gitelman syndrome. Biochemical criteria were also adequate to further raise our suspicion of Gitelman syndrome. Genetic testing for Gitelman syndrome was not done, due to the unavailability of genetic testing as well as funding for tests to be sent abroad.

Initially, hypokalemia was managed with an intravenous potassium supplement, hyperkalemia with oral free fluid, and hypomagnesaemia with an intravenous magnesium supplement. The patient was advised not to restrict fluid intake and to drink as per his thirst. He was prescribed oral potassium chloride and magnesium supplements. He was reviewed after 2 weeks with repeated serum electrolyte levels that were within normal limits, and his nighttime polyuria symptoms decreased. He was advised to check his serum electrolyte levels regularly. In a follow up visit after 2 months of discharge, his serum potassium levels were at a lower normal level, and was advised to increase intake of potassium rich diet.

## DISCUSSION

Nephrogenic diabetes insipidus is either congenital or acquired and includes chronic kidney diseases such as polycystic kidneys and obstructive uropathy; metabolic conditions such as hypokalemia and hypercalcaemia; drugs such as lithium and demeclocycline; osmotic diuresis such as glucose and mannitol; amyloidosis; and myelomatosis.^4^ In this case study, hypokalemia is the cause of nephrogenic diabetic insipidus. The most challenging part of diagnosing a suspected case of DI is deciding whether it is central DI, nephrogenic DI, or primary polydipsia. The water deprivation test, also known as the indirect water deprivation test, can potentially be able to differentiate among the different types of DI.^2^

The urine osmolality in partial NDI varies from 300 to 700 mOsm/kg, and desmopressin will cause a little increase in urine osmolality (up to 45%). In contrast, the reaction to desmopressin is either negligible or nonexistent in cases of total NDI, when urine osmolality is less than 300 mOsm/kg. Without prior thirsting (without water deprivation/hypertonic saline infusion), baseline plasma copeptin levels of ≥21.4 pmol/L or a plasma AVP level of ≥3 pg/ml have been shown to distinguish nephrogenic DI (partial and complete) from other types of polyuria-polydipsia syndromes with 100% sensitivity and specificity.^2^ We suspected nephrogenic diabetes insipidus as patient serum osmolarity was above normal limit and confirmed by water deprivation test as well as high plasma copeptin levels. Also, minimal response to desmopressin trial helped to diagnose partial nephrogenic DI.

Gitelman syndrome is an autosomal recessive genetic disorder that is due to a loss-of-function mutation in the gene SLCI2A3, which codes for the thiazide diuretic sensitive Non Contrast Computed Tomography (NCCT) found in the distal renal tubules. Aldosterone and renin levels rise, and the Renin Angiotensin Aldosterone System (RAAS) system is activated by reduced sodium chloride reabsorption, blood volume, and renal salinisation, which leads to hypokalemia and metabolic alkalosis.^5^ The condition is often diagnosed in adolescence or adulthood, and symptoms typically do not manifest before the age of six.^6^

The proposed biochemical criteria for suspecting Gitelman syndrome in a patient include the following; (1) Documented chronic hypokalemia (2.0 mmol/mmol [>18 mmol/g]) in the absence of potassium-lowering drugs; (2) Metabolic alkalosis; (3). Hypomagnesaemia; (4) Hypocalciuria (spot urine, calcium-creatinine ratio 0.5%; (5) Normal or low blood pressure; (6) Normal renal ultrasound with the absence of nephrocalcinosis or renal abnormalities.^3^ Hypokalemia causes autophagic degradation of aquaporin-2 and decreases the response to the antidiuretic hormone arginine vasopressin (AVP), resulting in nephrogenic diabetes insipidus.^7^ Treatment is usually symptomatic, which includes potassium and magnesium supplementation. Renin-angiotensin system blockers, potassium-sparing diuretics, nonsteroidal anti-inflammatory drugs (NSAIDs), like indomethacin, or a combination of these have been advised in cases of persistent or symptomatic hypokalemia, even despite supplementation, or when adverse reactions are unacceptable.^3^ In our case report, Gitelman syndrome was suspected based on biochemical criteria but couldn't be confirmed with genetic testing due it's unavailability.

We hereby present the rare case of partial nephrogenic diabetes insipidus owing to hypokalemia with Gitelman syndrome meeting biochemical and clinical criteria. To the best of our knowledge, no such cases have been reported to date. Furthermore, the treatment of hypokalemia resulted in the reversal of partial nephrogenic diabetes insipidus in this case study.

## CONCLUSIONS

Our case presented with partial nephrogenic diabetes insipidus owing to hypokalemia. Given the rarity of nephrogenic diabetes insipidus associated with Gitelman syndrome, it is quite difficult to correlate in clinical settings. However, if the patient presents with polyuria and polydipsia syndrome in the setting of hypokalemia, an evaluation of hypokalemia and nephrogenic DI should be performed. Furthermore, the treatment of hypokalemia resulted in the reversal of partial nephrogenic diabetes insipidus in this case study. To determine whether correction of hypokalemia can reverse nephrogenic diabetes insipidus, more case reports and studies are required.

Due to the unavailability of genetic testing for Gitelman syndrome in Nepal and the lack of funding to send samples to another country, the diagnosis couldn't be confirmed.
